# TFPIα and TFPIβ are expressed at the surface of breast cancer cells and inhibit TF-FVIIa activity

**DOI:** 10.1186/1756-8722-6-5

**Published:** 2013-01-15

**Authors:** Benedicte Stavik, Mari Tinholt, Marit Sletten, Grethe Skretting, Per Morten Sandset, Nina Iversen

**Affiliations:** 1 Department of Medical Genetics, Oslo University Hospital and University of Oslo, BOX 4956 Nydalen, Oslo, N-0424, Norway; 2 Department of Haematology, Oslo University Hospital, BOX 4956 Nydalen, Oslo, N-0424, Norway; 3 Research Institute of Internal Medicine, Oslo University Hospital, BOX 4956 Nydalen, Oslo, N-0424, Norway; 4 Institute of Clinical Medicine, University of Oslo, Oslo, Norway

**Keywords:** TFPIα, TFPIβ, TF-FVIIa, Breast cancer cells, Endothelial cells, Heparin, PI-PLC, Anticoagulant activity

## Abstract

**Background:**

Tissue factor (TF) pathway inhibitor-1 (TFPI) is expressed in several malignant tissues- and cell lines and we recently reported that it possesses anti-tumor effects in breast cancer cells, indicating a biological role of TFPI in cancer. The two main splice variants of TFPI; TFPIα and TFPIβ, are both able to inhibit TF-factor VIIa (FVIIa) activity in normal cells, but only TFPIα circulates in plasma. The functional importance of TFPIβ is therefore largely unknown, especially in cancer cells. We aimed to characterize the expression and function of TFPIα, TFPIβ, and TF in a panel of tumor derived breast cancer cell lines in comparison to normal endothelial cells.

**Methods:**

TFPIα, TFPIβ, and TF mRNA and protein measurements were conducted using qRT-PCR and ELISA, respectively. Cell-associated TFPI was detected after phosphatidylinositol-phospholipase C (PI-PLC) and heparin treatment by flow cytometry, immunofluorescence, and Western blotting. The potential anticoagulant activity of cell surface TFPI was determined in a factor Xa activity assay.

**Results:**

The expression of both isoforms of TFPI varied considerably among the breast cancer cell lines tested, from no expression in Sum149 cells to levels above or in the same range as normal endothelial cells in Sum102 and MDA-MB-231 cells. PI-PLC treatment released both TFPIα and TFPIβ from the breast cancer cell membrane and increased TF activity on the cell surface, showing TF-FVIIa inhibitory activity of the glycosylphosphatidylinositol- (GPI-) anchored TFPI. Heparin treatment released TFPIα without decreasing the cell surface levels, thus indicating the presence of intracellular storage pools of TFPIα in the breast cancer cells.

**Conclusion:**

GPI-attached TFPI located at the surface of breast cancer cells inhibited TF activity and could possibly reduce TF signaling and breast cancer cell growth locally, indicating a therapeutic potential of the TFPIβ isoform.

## Introduction

Tissue factor (TF) pathway inhibitor-1 (TFPI) is a plasma serine protease inhibitor, which is mainly known for its role in the coagulation cascade, being responsible for the modulation of TF induced blood coagulation. The human *TFPI* gene is positioned on chromosome 2 and spans about 70kb [1,2]. Two main splice variants are transcribed from *TFPI*; TFPIα and TFPIβ [1,3,4]. Both mRNAs encode a signal peptide that redirects the translating ribosome to the ER, inserting the protein into the ER lumen as it is translated. The proteins are post-translationally modified in the ER and in Golgi, and are then transported by vesicles to the exterior of the cell [5]. The mature TFPIα protein comprises 276 amino acids and contains an N-terminal end, three Kunitz-type inhibitor domains, and a positively charged C-terminal tail [1]. Intracellular TFPIα is thought to either remain soluble or bind to an unknown co-factor containing a glycosylphosphatidylinositol (GPI) anchor [5]. Depending on the nature of TFPIα inside the cell, the protein is either secreted or indirectly attached to the outer cell wall after transportation to the outer cell membrane [5,6]. In comparison, the mature TFPIβ protein comprises 223 amino acids. It shares amino acids 1–181 with TFPIα, and thus the N-terminal end and the first two Kunitz-type domains, but has a different C-terminal end containing a GPI attachment signal that exclusively localizes it to the cell membrane [6].

*In vivo*, only TFPIα is detected in circulating blood and this isoform is therefore considered to be important in arresting the coagulation initiation. Heparin treatment rapidly increase the release of TFPI from endothelial cells both *in vivo*[7] and *in vitro*[8,9], which may be an important anticoagulant effect of heparins [10,11]. Cell-associated TFPIα and TFPIβ are both able to inhibit the activity of the TF-factor VIIa (FVIIa) catalytic complex in normal cells *in vitro*[12] although their contribution to the anticoagulant effect is not completely understood. In cancer, TF expression and function has been extensively studied and the non-haemostatic effects of TF in promoting cancer cell metastasis and angiogenesis are well documented [13-19]. In contrast, TFPI has shown anti-tumor effects. We recently investigated the biological relevance of TFPI in breast cancer cells through overexpression and knockdown studies and found that both isoforms exerted tumor suppressing properties, such as increased apoptosis and reduced proliferation- and migration/invasion *in vitro*[20-22]. Furthermore, several cancer cell lines and tumors have been reported to express TFPIα [23,24], and enhanced levels of both TFPIα and TFPI-factor Xa (FXa) complexes have been detected in plasma of patients with solid tumors [25,26], supporting that TFPI may be involved in malignant disease mechanisms.

To further explain the role of TFPI in malignant disease, we have in the present study aimed to characterize TFPI by investigating the expression and localization of TFPIα and TFPIβ, and also the relation to TF expression and activity, in a panel of breast cancer cell lines. Since the endothelium is considered to be the predominant site of TFPI synthesis, the TFPI characteristics of the breast cancer cells were compared to normal endothelial cells. The selected breast cancer cell lines have been derived from different breast tumors, and are classified as basal- or luminal-like, and primary or metastatic according to the subtype and origin of the tumor, respectively. We report here considerable variations in the expression of both TFPI isoforms among the various breast cancer cell lines tested. The expression ranged from not detected to levels comparable to and even higher than observed in normal, primary endothelial cells. TFPIα was detected in the cell medium and on the surface of breast cancer cells, attached through a GPI anchor, while TFPIβ was exclusively located on the cell surface. The GPI-attached, cell bound TFPI was found to be functionally active, and to our knowledge, demonstrating for the first time anticoagulant properties of TFPI expressed on the surface of breast cancer cells. Heparin treatment increased the TFPIα levels in the supernatant without altering the cell surface levels, indicating the presence of intracellular storage pools of TFPIα in the breast cancer cells.

## Results

### TFPIα and TFPIβ mRNA and protein expression levels

**Figure 1:**
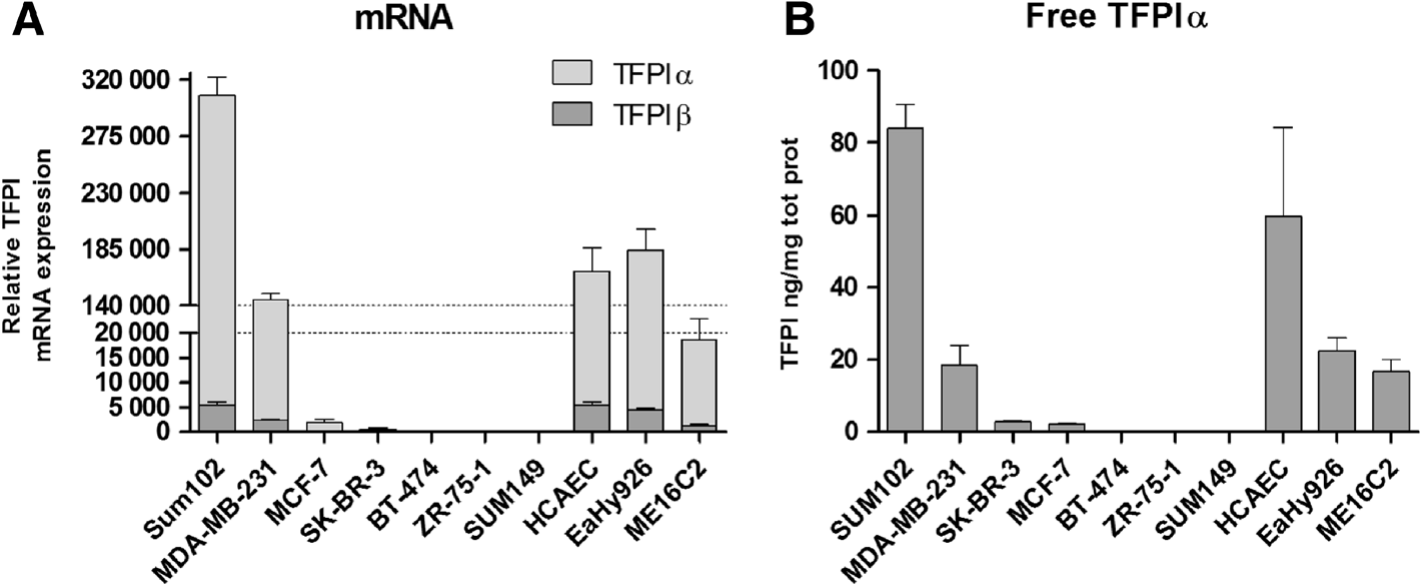
**TFPI expression in different breast cancer cell lines and normal cells.** Cells were seeded in 6-wells trays to reach ~80% confluence in three days before media were collected and cells lysed for RNA isolation. (**A**) Relative TFPIα and TFPIβ mRNA expression measured using qRT-PCR. ΔΔCt values were calculated using 18s rRNA as an endogenous control and the TFPI negative, non-human CHO cells as a negative control. (**B**) Secreted TFPIα protein measured in cell media and normalized to total protein amounts. Mean values + SD (n = 3) are presented.

Quantitative real-time PCR using reverse primers specific for TFPIα and TFPIβ was performed to determine the relative mRNA expression of the two isoforms in the various breast cancer cell lines. The results were normalized against the TFPI expression level in the primary human coronary artery endothelial cells (HCAECs) (Table 1), while the TFPI negative non-human Chinese hamster ovary (CHO) cell line was used as a negative control in Figure 1A. The breast cancer cell lines expressed TFPIα mRNA in the following high to low order: Sum102 > MDA-MB-231 > MCF-7 > SK-BR-3 > BT-474 > ZR-75-1 > Sum149. TFPIβ mRNA was expressed in the same high to low order as TFPIα (Table 1), and the expression of the two isoforms correlated significantly (r = 0.94, *p* < .001). As illustrated in Figure 1A, the Sum102 breast cancer cells expressed twice as much TFPIα and β mRNA as the MDA-MB-231 cells, and 17- and 4-fold more TFPIα and TFPIβ mRNA, respectively, than the non-cancerous breast epithelial cell line ME16C2. The Sum102 cell line expressed twice as much TFPIα and similar levels of TFPIβ as the HCAECs and the endothelial cell line EA.hy926, while the MDA-MB-231 cells expressed similar levels of TFPIα, but only half the amount of TFPIβ as the HCAECs and EA.hy926 cells (Table 1 and Figure 1A). Compared to the HCAECs, the relative TFPIα mRNA expression was 10-fold lower in the non-cancerous breast epithelial cells ME16C2 and 100 – 1000-fold lower in the breast cancer cell lines SK-BR-3 and MCF-7 (Table 1). Virtually no TFPIα or TFPIβ mRNA was expressed by the BT-474, ZR-75-1, and the Sum149 cell lines (Figure 1A and Table 1).

**Table 1:** Characterization of TFPI and TF in a selection of tumor derived breast cancer cell lines and normal cells

**Cell types**	**Description/Subtype**	**Origin**	**Invasiveness **(***In vitro***)*	**TFPIα mRNA (RQ)**	**TFPIβ mRNA (RQ)**	**Relative Free TFPIα (secreted)**	**TF mRNA (RQ)**	**Relative fl**^**† **^**TF (cell-associated)**
SUM102	Breast epithelial, basal-like	Tumor (primary)	Invasive	1.85	1.02	1.41	129	105
MDA-MB-231	Breast epithelial, basal-like	Tumor (metastatic)	Invasive	0.87	0.46	0.31	894	705
MCF-7	Breast epithelial, luminal-like	Tumor (metastatic)	Non-invasive	0.01	0.02	0.04	0.45	0.96
SK-BR-3	Breast epithelial, luminal-like	Tumor (metastatic)	Non-invasive	3E-03	0.02	0.05	1.58	1.64
BT-474	Breast epithelial, luminal-like	Tumor (primary)	Non-invasive	8E-04	6E-03	n.d	2.63	1.32
ZR-75-1	Breast epithelial, luminal-like	Tumor (metastatic)	Non-invasive	7E-04	5E-03	n.d	3.55	2.33
SUM149	Breast epithelial, basal-like	Tumor (primary)	Invasive	2E-04	4E-03	n.d	4373	948
HCAEC	Coronary artery endothelial	Primary (normal)	Non-invasive	1.00	1.00	1.00	1.00	1.00
EaHy926	Umbilical vein endothelial	Transformed	Non-invasive	1.11	0.83	0.38	0.62	0.61
ME16C2	Breast epithelial, basal-like	Primary (immortalized)	Invasive	0.11	0.25	0.28	336	170

* from references [38-42].

RQ - relative quantification calculated using 18sRNA as endogenous control. All values were calculated against HCAEC expression as a normal endothelial reference.

^†^ full length.

n.d. - not detected, below lowest standard.

TFPI and TF mRNA and protein levels were measured using qRT-PCR and ELISA in the breast cancer cells and in the non-cancerous breast epithelial cells ME16C2 and umbilical vein endothelial cell line EaHy926. All values were compared to the primary human coronary artery endothelial cells HCAEC and presented as relative values, mRNA results by the comparative Ct method and antigen levels by direct comparison.

TFPI antigen levels were measured using the free and total TFPI enzyme-linked immunoabsorbent assay (ELISA) kits. The free TFPI kit detects an epitope in the third Kunitz domain of TFPI and thereby measure only TFPIα. The total TFPI kit detects an epitope between the first two Kunitz domains and it therefore able to measure both TFPI isoforms. ELISA of recombinant TFPI_1-161_, which lacks the third Kunitz domain, confirmed this, as only the total, but not the free kit detected the truncated TFPI (data not shown). Secreted TFPIα was measured in the cell medium, and TFPIα mRNA and protein levels correlated significantly (r = 0.94, *p* = .002). The breast cancer cell lines secreted TFPIα in the following high to low order: Sum102 > MDA-MB-231 > MCF-7 and SK-BR-3. No detectable levels of TFPIα protein were secreted by the BT-474, ZR-75-1, and Sum149 cells (Figure 1B and Table 1). The Sum102 cell line secreted 40% more TFPIα than the HCAECs, while the MDA-MB-231 cells secreted TFPIα levels within the range of the endothelial cell line EA.hy926 and the non-cancerous breast epithelial cells ME16C2 (Figure 1B and Table 1). The breast cancer cell lines that expressed abundant TFPIα and TFPIβ originated from both primary and metastatic basal-like tumors and have previously been shown to display invasive characteristics *in vitro* (Table 1).

### TF mRNA and antigen

TF protein levels were measured in the cell lysate and correlated significantly with mRNA expression in all the cell lines (r = 0.99, *p* < .001). Moreover, the expression of TF associated with TFPI expression in all breast cancer cell lines tested except the Sum149 cells. Compared to the endothelial cells HCAEC, high levels of TF mRNA were detected in the basal-like, invasive breast cancer cell lines Sum102, MDA-MB-231, and Sum149, and also in the non-cancerous breast epithelial cells ME16C2. In contrast, the luminal-like, non-invasive breast cancer cell lines MCF-7, SK-BR-3, BT-474, and ZR-75-1 expressed low levels of TF mRNA, in the same range as the endothelial cells HCAEC and EA.hy926 (Table 1). The breast cancer cells Sum149 and MDA-MB-231 expressed 13- and 3-fold more TF mRNA, respectively, compared to the non-cancerous ME16C2 cells, while the Sum102 cell line expressed less TF mRNA relative to ME16C2 cells, but 130-fold more than the HCAECs (Table 1).

### Cell-associated TFPI in breast cancer cells versus normal endothelial cells

**Figure 2:**
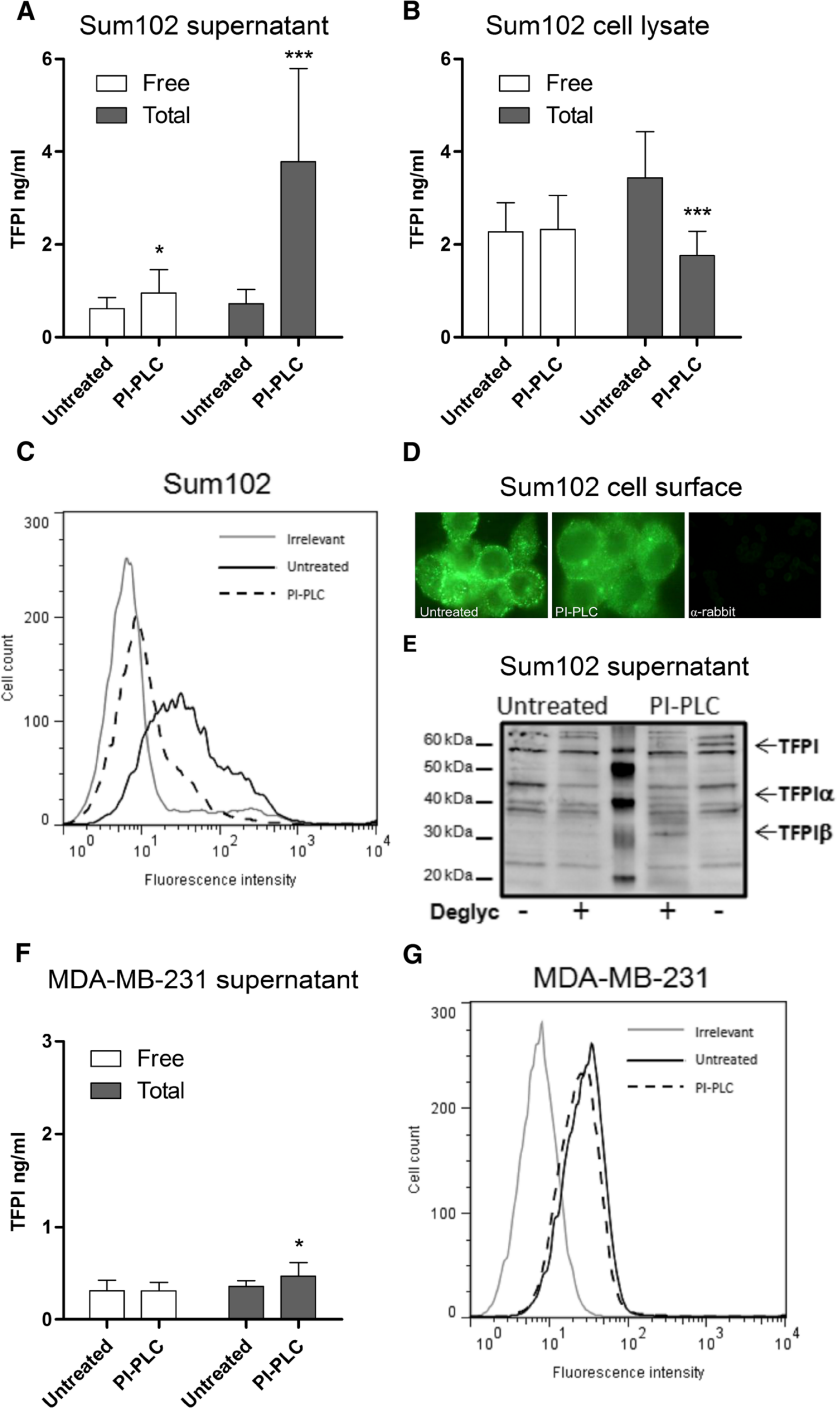
**PI-PLC treatment of Sum102 and MDA-MB-231 cells.** Serum-starved cells were treated with SFM (untreated) or PI-PLC (1 U/mL) for 2 hours before the supernatant was removed and cells lysed or collected for flow cytometry analysis. Total and free TFPI antigen measured in the supernatant (**A** and **F**) and lysate (**B**) of untreated and PI-PLC treated Sum102 (**A** and **B**) and MDA-MB-231 (**F**) cells. Mean values + SD (n ≥ 8) of three to six independent experiments are presented. All statistical comparisons were conducted between PI-PLC treated samples and untreated controls. Flow cytometry analysis of untreated (black, solid) and PI-PLC treated (black, broken) Sum102 (**C**) and MDA-MB-231 (**G**) cells. One representative experiment of three is shown. (**D**) Fluorescence images of SFM + glycerol (untreated, left panel) and PI-PLC (middle panel) treated Sum102 cells stained with antibody against both isoforms of TFPI. The right panel shows a secondary antibody background control staining. One representative experiment of three is shown. (**E**) Western blot of supernatants from SFM + glycerol (untreated) and PI-PLC treated Sum102 cells. Samples were deglycosylated and incubated with a polyclonal anti-human TFPI antibody.

The breast cancer cell lines Sum102 and MDA-MB-231, possessing high TFPI expression, were further analyzed to determine the nature of any cell-associated TFPI. Cleavage of the GPI anchors by phosphatidylinositol-phospholipase C (PI-PLC) treatment resulted in a significant 1.5- and 5.3-fold increase in free and total TFPI antigen levels, respectively, in the Sum102 cell supernatant (Figure 2A), and a corresponding 49% decrease in total, but not free TFPI antigen levels, in the cell lysate (Figure 2B). PI-PLC treatment reduced the amount of TFPI positive Sum102 cells from 31.8 (± 3.9) to 3.9 (± 0.2) as measured by flow cytometry, corresponding to an approximately 75% removal of the cell surface TFPI (Figure 2C). Immunostaining demonstrated a clear, but not a complete removal of cell surface TFPI after PI-PLC treatment of the cells (Figure 2D). This effect was also apparent when a TFPIα specific antibody was used (data not shown). Western blot analysis of deglycosylated PI-PLC supernatants showed that both isoforms of TFPI were released after PI-PLC treatment of Sum102 cells (Figure 2E). Slightly heavier bands than previously reported for TFPI was observed, which may be due to cancer cell specific modifications. No significant increase in free and a minor increase in total TFPI antigen was detected in the MDA-MB-231 cell supernatants after PI-PLC treatment (Figure 2F), and a slight reduction in cell surface TFPI levels was seen by flow cytometry (Figure 2G). No significant decrease in free or total TFPI was detected in the cell lysate of these cells after PI-PLC treatment (data not shown).

**Figure 3:**
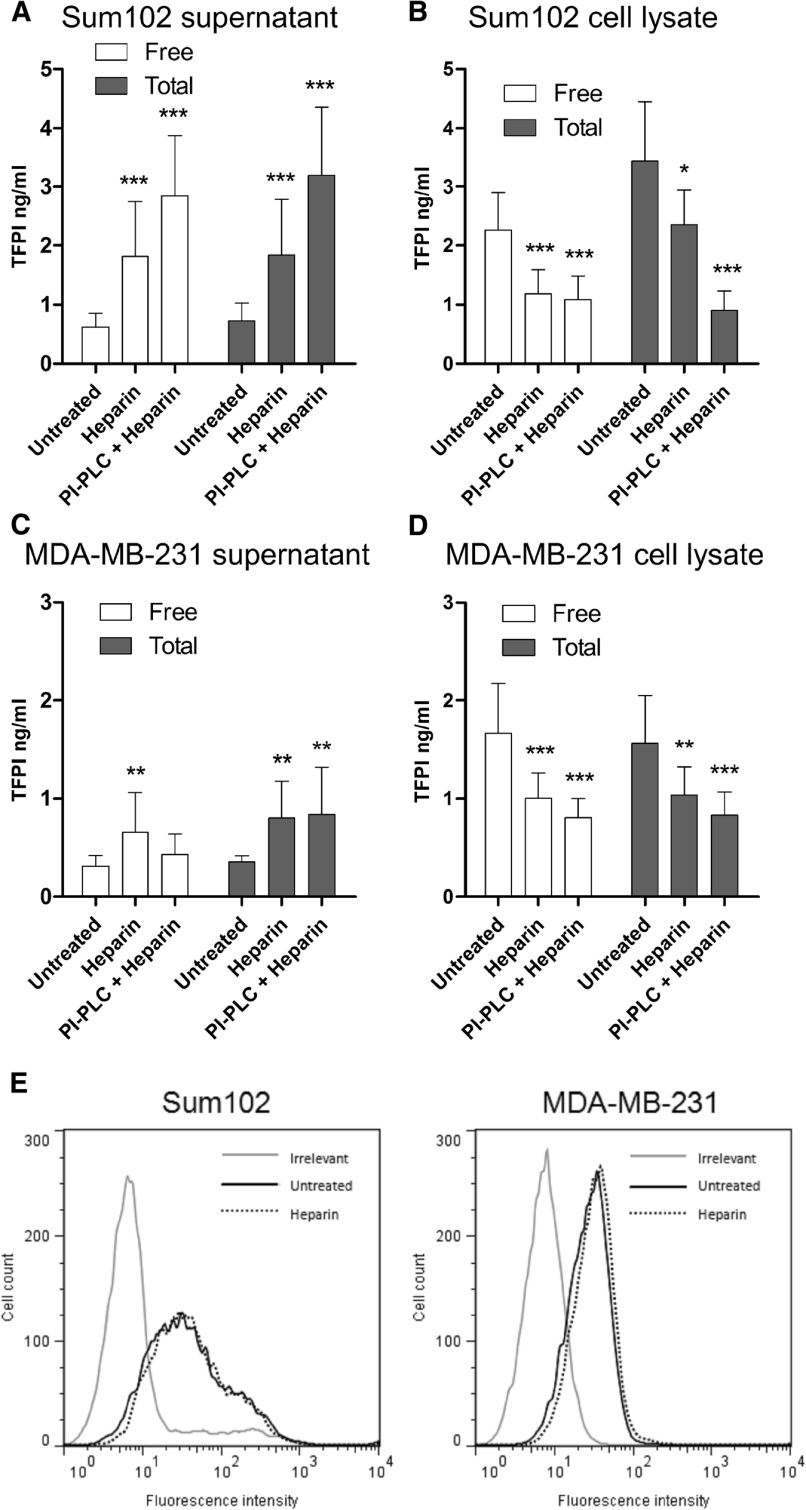
**Heparin treatment of Sum102 and MDA-MB-231 cells.** Serum-starved cells were treated with SFM (untreated) or heparin (5 U/mL) for 2 hours or with PI-PLC + heparin (2+2 hours) before the supernatant was removed and cells lysed or collected for flow cytometry analysis. The supernatant was discarded after PI-PLC pre-treatment. Free and total TFPI antigen measured in the supernatant (**A** and **C**) and lysate (**B** and **D**) of untreated, heparin or PI-PLC + heparin treated Sum102 (**A** and **B**) and MDA-MB-231 (**C** and **D**) cells. Mean values + SD (n ≥ 9) of three to six independent experiments are presented. Statistical comparisons were conducted between heparin/PI-PLC+heparin treated samples and untreated controls. (**E**) Flow cytometry analysis of untreated (black, solid) and heparin treated (black, dotted) Sum102 (left panel) and MDA-MB-231 (right panel) cells. One representative experiment of three is shown.

Heparin treatment of Sum102 cells increased the free and total TFPI antigen levels 2.9- and 2.6-fold, respectively, in the cell supernatant (Figure 3A). A corresponding 48% and 32% decrease in free and total TFPI, respectively, was seen in the cell lysate (Figure 3B). PI-PLC pre-treatment prior to heparin treatment resulted in an additional 60-70% increase in free and total TFPI antigen levels in Sum102 supernatant compared to heparin treatment alone (Figure 3A). A reduction in TFPI levels was also observed in cell lysates, especially apparent in the total TFPI levels (Figure 3B). A 2.1- and 2.3-fold increase in free and total TFPI antigen levels, respectively, was observed in the supernatant of MDA-MB-231 cells after heparin treatment, though no additional increase was seen when the cells were pre-treated with PI-PLC (Figure 3C). Heparin reduced free and total TFPI levels in the cell lysate by 40% and 34%, respectively, and pre-treatment with PI-PLC reduced the levels even further (Figure 3D). Flow cytometry showed no reduction of cell surface TFPI levels after heparin treatment in either cell line (Figure 3E), and no additional reduction in cell surface TFPI was observed after heparin treatment of PI-PLC pre-treated cells (data not shown).

**Figure 4:**
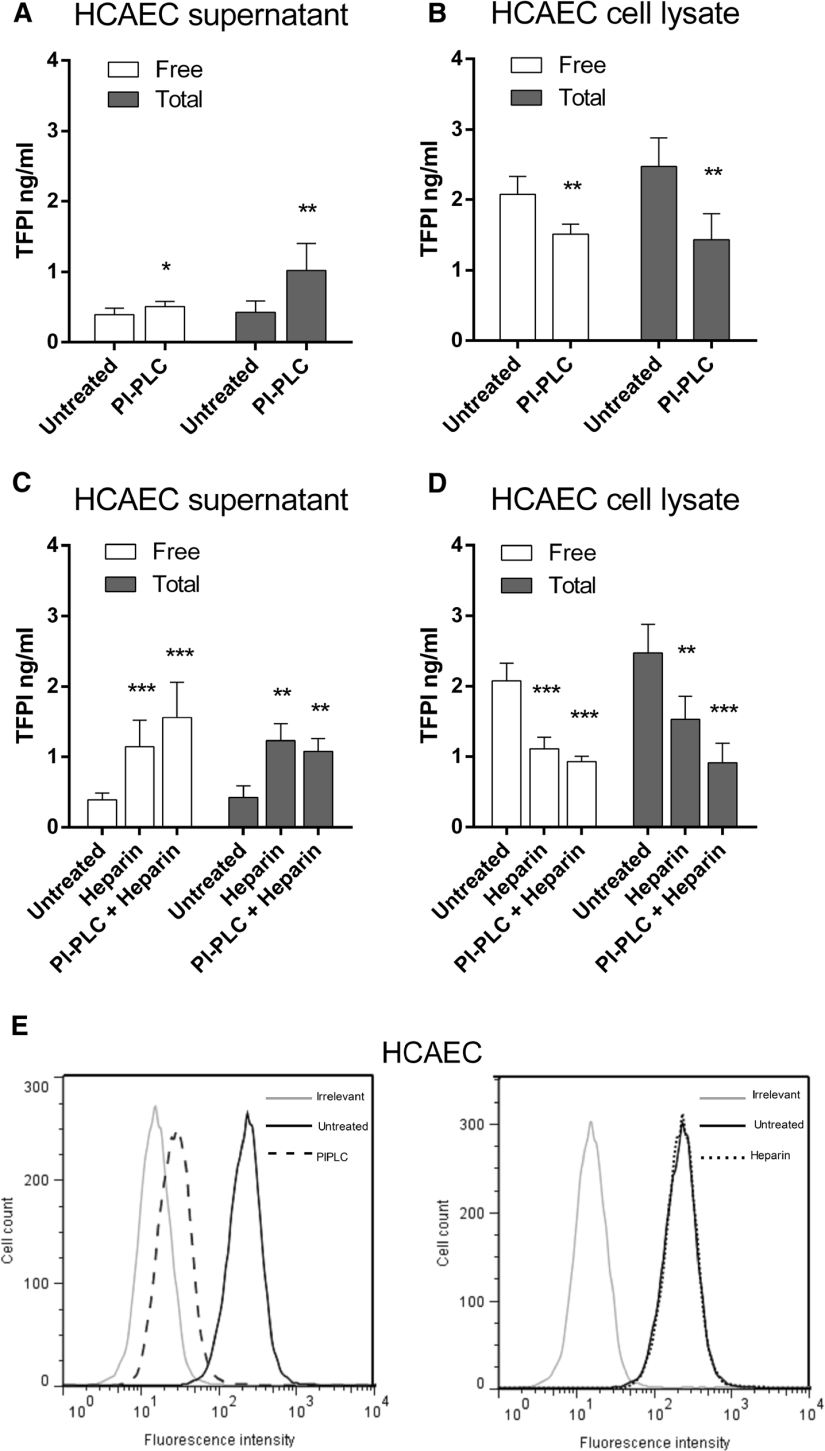
**PI-PLC and heparin treatment of HCAEC cells.** Serum-starved cells were treated with SFM (untreated), PI-PLC (1 U/mL), or heparin (5U/mL) for 2 hours or with PI-PLC + heparin (2+2 hours) before the supernatant was removed and cells lysed or collected for flow cytometry analysis. The supernatant was discarded after PI-PLC pre-treatment. Total and free TFPI antigen measured in the supernatant (**A** and **C**) and cell lysate (**B** and **D**) of untreated/PI-PLC (**A** and **B**) and untreated/heparin/PI-PLC+heparin (**C** and **D**) treated HCAEC cells. Mean values (n ≥ 5; supernatant, n = 4; cell lysate) + SD of two independent experiments. Statistical comparisons were conducted between PI-PLC or heparin/PI-PLC+heparin treated samples and untreated controls. (**E**) Flow cytometry analysis of untreated (black, solid), PI-PLC (black, broken, left panel), and heparin (black, dotted, right panel) treated HCAEC cells. One representative experiment of three is shown.

The TFPI characteristics in the breast cancer cell lines were very similar to those observed in the primary endothelial cells HCAEC. In the supernatant, free and total TFPI antigen levels were increased 1.3- and 2.4-fold, respectively (Figure 4A) after PI-PLC treatment. Complementary, the free and total TFPI antigen levels in the cell lysate were reduced by 27% and 40%, respectively (Figure 4B). Measured by flow cytometry, TFPI positive cells were reduced from 200.4 (±16.9) to 43.1 (±1.9) following PI-PLC treatment, corresponding to a 90% removal of TFPI from the endothelial cell surface (Figure 4E, left panel). As demonstrated for Sum102 and MDA-MB-231 cells, no reduction in cell surface TFPI was observed by flow cytometry after heparin treatment of HCAECs (Figure 4E, right panel), though total TFPI levels were increased 2-fold in the cell supernatant and reduced by 38% in the cell lysate. Furthermore, free TFPI was increased 2.9-fold in the supernatant and reduced by 46% in the cell lysate (Figure 4C and D). Sequential treatment with PI-PLC followed by heparin did not reduce TFPI cell surface levels any further than PI-PLC alone (data not shown), which is equivalent to the observations in the breast cancer cell lines. Moreover, PI-PLC pre-treatment of HCAEC cells followed by heparin released 50% more free TFPI into the supernatant and reduced cell lysate levels additionally compared to heparin alone (Figure 4C and D) as seen in the Sum102 breast cancer cells.

### TF-FVIIa activity and co-localization

**Figure 5:**
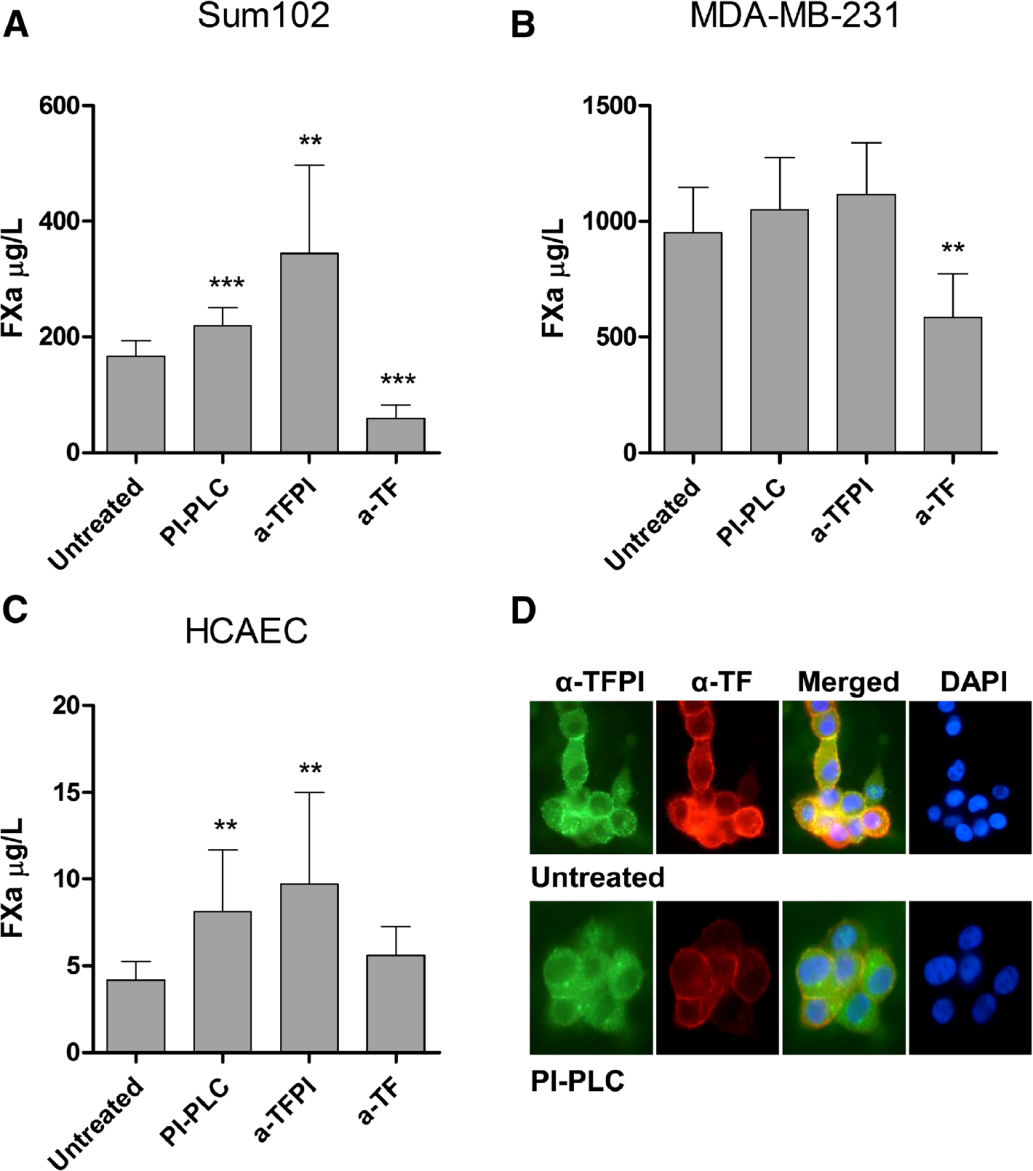
**Cell surface anticoagulant activity of PI-PLC treated cells.** Confluent Sum102, MDA-MB-231, and HCAEC cells grown in 24-wells trays were treated with SFM (untreated), PI-PLC, monoclonal anti-TFPI antibody, or anti-TF antibody for 2 hours before the supernatant was removed and cells were washed and assayed for TF activity. Sum102 (**A**), MDA-MB-231 (**B**), and HCAEC (**C**) cells were incubated with FVIIa and FX for 1 hour before the reaction was stopped and a substrate to FXa was added. The results were analyzed colorimetrically at 405 nm. The amount of FXa generated is displayed as mean mU/mL + SD (n ≥ 7) of three to four independent experiments. Statistical comparisons were conducted between PI-PLC treated samples and untreated controls. (**D**) Fluorescence images of SFM + Glycerol (untreated, upper panels) and PI-PLC (bottom panels) treated Sum102 cells stained with antibodies against both isoforms of TFPI (green, left panels) and TF (red, middle-left panels). After merging the images, co-localization of the two antibodies was indicated by a yellow color (middle-right panels). Nuclear staining with DAPI is depicted in the left panels. One representative experiment of three is shown.

To explore the potential anticoagulant activity of the breast cancer and endothelial cell-associated TFPI, we measured the TF activity on the Sum102, MDA-MB-231 and HCAEC cell surface indirectly by a chromogenic FXa activity assay. The generation of FXa on the surface of Sum102 and HCAEC cells increased significantly by 1.3- and 2-fold, respectively, following PI-PLC treatment (Figure 5A and C). A small 10% increase in FXa levels was observed on MDA-MB-231cells after PI-PLC treatment, although not significant probably due to the abundant TF levels in these cells (Figure 5B). Treating cells with anti-TFPI blocking antibody resulted in higher FXa activity than PI-PLC treatment in all cells tested (Figure 5A-C), indicating that additional non-GPI bound TFPI with anticoagulant activity is present on the cell surface. ELISA results confirmed that PI-PLC treatment did not alter the TF antigen levels in the cells compared to untreated controls (data not shown). Control experiments performed in the absence of FVIIa showed no generation of FXa (data not shown), and the addition of anti-TF blocking antibody prior to analysis suppressed the FXa generation significantly, except in the HCAEC cells (Figure 5A-C). Immunostaining with both anti-TFPI and anti-TF antibodies simultaneously demonstrated a co-localization of these proteins at the surface of Sum102 cells (Figure 5D, upper panels), which was diminished after PI-PLC treatment (Figure 5D, bottom panels).

## Discussion

In the present study, we have characterized the mRNA and protein expression of two isoforms of TFPI (α and β) and also TF, in a panel of breast cancer cell lines in comparison to normal endothelial cells. We have provided novel evidence that non-cancerous and malignant breast epithelial cells express TFPIβ protein. The breast cancer cell lines are classified as either basal- or luminal-like according to the subtype of the tumor they were derived from. In general, basal-like breast cancer cells tend to lack hormone receptors and are more invasive than luminal-like cells [27,28]. The TFPI expression seemed to correlate well with invasiveness and basal-like origin of the cells, except for the TFPI negative Sum149 cell line. Among the cell lines tested, TFPI expression was detected in the following high to low order; Sum102 > MDA-MB-231 > MCF-7 > SK-BR-3 > BT-474 > ZR-75-1 > Sum149, which is comparable to data from Kao and co-workers [28] when we extracted the TFPI expression results from their publicly available data file of gene expression profiles of different breast cancer cell lines. The TFPI expression also seemed to correlate with the growth rate of the cells as MDA-MB-231, MCF-7, BT-474, and ZR-75-1 have a doubling time of approximately 23, 29, 72, and 80 hours, respectively. Moreover, high expression of TF was also observed in cells expressing TFPI which may contribute to the growth of the cells. Previously, the MDA-MB-231 cells were also shown to express TFPI mRNA and protein [23]. The relative mRNA ratios between the two TFPI isoforms correlated well in all the breast cancer cell lines in terms of TFPIα being the major isoform, which is consistent with our findings and previous results in endothelial cells [6]. A positive correlation between TFPIα mRNA and secreted antigen levels was observed in the cell lines. However, HCAEC cells secreted 3-fold more TFPIα protein than MDA-MB-231 and EA.hy926 cells, although TFPIα mRNA levels were similar. This could imply that less TFPIα is cell-associated in these cells compared to the MDA-MB-231 breast cancer and EA.hy926 endothelial cells.

Recently, Girard and co-workers reported that TFPIβ was the sole TFPI isoform associated with the SK-Hep-1 endothelial cell surface through a GPI anchor [29]. In contrast to this, our findings indicated that both TFPIα and TFPIβ were attached to the surface of the Sum102 breast cancer cells through GPI anchors as confirmed by ELISA and Western blotting of deglycosylated PI-PLC treated supernatants. Moreover, a significant increase in both free and total TFPI was observed after PI-PLC treatment of the normal endothelial cells HCAEC in this study, although ELISA results indicated that TFPIβ was the most abundant GPI-attached isoform in both cell types. Endothelial cells have previously been shown to express TFPIα on the cell surface [30], and in a study by Piro and Broze the endothelial cells ECV304 (later identified as a bladder carcinoma cell line [31]) were shown to express both TFPIα and TFPIβ on the surface [12]. These results, together with our findings, may indicate cell type dependent differences in the expression of a GPI-attached cofactor for TFPIα. Intriguingly, no reduction in free TFPI was observed in Sum102 cell lysates after PI-PLC treatment as seen in the HCAEC cell lysates. This may be due to higher background levels of free TFPI in the Sum102 cell lysate than in the supernatant. Thus it seems likely that the amount of GPI-attached TFPIα was insignificant compared to the total amount of cell-associated TFPIα in these cells. Low levels of TFPI seemed to be released from the MDA-MB-231 cells after PI-PLC treatment, which may be due to the relative low TFPIβ expression in these cells compared to the Sum102 and endothelial cells, or to other structural differences on the surface of these cells that made the GPI anchors inaccessible to PI-PLC.

Heparin treatment resulted in the release of TFPI from the Sum102 and MDA-MB-231 breast cancer cells in a similar manner to that observed in normal endothelial cells in this and a previously study [32]. Moreover, our findings support the growing evidence that the full length TFPIα is the exclusive TFPI isoform released upon heparin treatment as similar levels were detected using either ELISA kit. Heparin treatment released TFPI from breast cancer cells without reducing cell surface levels, which is consistent with what was observed in endothelial cells in this and other studies [6,8,9,30]. Thus, our findings indicate that the breast cancer cells possessed intracellular storage pools of TFPIα in a similar manner to that suggested for normal endothelial cells. The basic mechanism and exact cellular localization of this heparin-releasable TFPI is, however, currently unknown. In contrast to what was reported previously for HUVECs [32], serum was not necessary for the release of TFPIα by heparin in the breast cancer cells or HCAECs, although serum did increase the amount of TFPI released by heparin. Treatment with PI-PLC uncovered a heparin-releasable TFPI pool that was not accessible before treatment, since PI-PLC prior to heparin treatment released additional TFPIα from the Sum102 and HCAEC cells. Thus, some GPI-attached cell surface protein(s) seemed to interfere with the ability of heparin to release intracellular TFPIα. This is in contrast to the findings of Ellery and co-workers in HUVECs [32], but consistent with those observed by Zhang et al. [6] and Mast and colleagues [33] in HUVEC, ECV406, and EA.hy926 cells and in human placenta. Contradictory to a previous report on endothelial cells [6] we did not observe any additional release of cell surface attached TFPI by heparin after PI-PLC pre-treatment in our cells. Our methodology is virtually the same, and it is therefore likely that this discrepancy may be due to yet unrevealed cell type specific characteristics like differences in the expression of other TFPI binding cell surface molecules such as syndecans [34].

When looking further into the characteristics of the different breast cancer cell lines, we discovered a similar substantial variation in the TF mRNA expression as observed for TFPI. The MDA-MB-231 and MCF-7 cells have previously been shown to express high and low levels of TF [35], respectively, which support our findings. Interestingly, the TF mRNA and antigen levels correlated well with the subtype and degree of invasiveness of the cells, supporting the tumor growth promoting nature of TF [13-18,36,37]. In accordance with the expression levels, the TF activity was greater in MDA-MB-231 cells than in Sum102 cells reflecting that TF was in fact more abundant on the surface of these cells. Moreover, the low TF expression in the HCAEC cells was reflected by the low levels of FXa generated in the assay and the inability to further reduce FXa activity after TF blocking. The increase in TF activity following PI-PLC treatment of Sum102 and HCAEC cells also indicated that the GPI-attached, cell surface TFPI was able to inhibit TF-FVIIa and/or FXa activity on the same cell. The immunostaining results also confirmed association of TFPI and TF at the cell surface. Thus, GPI-attached TFPI expressed by breast cancer cells was able to directly interfere with the TF activity on the cell surface and may therefore have implications for the pro-cancer nature of TF. Although we were unable to differentiate between TFPIα and TFPIβ in the procoagulant assay, TFPIβ appeared to be the most abundant isoform on the cell surface and we have previously observed that overexpression of TFPIα and TFPIβ separately suppressed breast cancer cell growth by inhibiting cell proliferation and increasing apoptotic activity [20], indicating functional importance of both isoforms. However, it remains to clarify whether the GPI-attached TFPI is indeed able to inhibit TF signaling, which is important in the pro-tumor effect of TF [18], or if the effects are due to signaling mechanisms independent of TF. Overexpression of TFPI in breast cancer cells conducted in previous experiments resulted in an increase in TF, PAR-1, and PAR-2 mRNA levels, which may indicate that TFPI is in fact able to affect TF signaling [20]. Pre-treatment with anti-TFPI blocking antibody increased TF activity even more than PI-PLC treatment, indicating the presence of non-GPI-attached cell surface TFPI that might possess anticoagulant activity.

## Conclusions

We provide evidence for the expression of both isoforms of TFPI (TFPIα and TFPIβ) in tumor derived breast cancer cell lines. The TFPI expression associated to some extent with the TF expression and the invasiveness and basal- or luminal-like subtype of the cells. TFPIα was secreted by the cells or remained cell-associated either bound to the surface indirectly through a GPI anchor or located in intracellular, heparin-releasable pools. TFPIβ was exclusively found on the cell surface attached through a GPI anchor. PI-PLC-releasable, GPI-attached cell surface TFPI co-localized with TF and inhibited TF-FVIIa activity and the removal of such anchored proteins allowed for the additional release of intracellular TFPIα by heparin. Furthermore, flow cytometry, immunofluorescence, and FXa activity measurements suggested the presence of a non-GPI bound cell surface associated TFPI pool with anticoagulant properties. Similar complex features of the two isoforms of TFPI were observed in breast cancer cells and in endothelial cells, although the variation in TFPI levels in the different breast cancer cell lines demonstrated a tumor-associated regulative control of the endogenous TFPI expression. Our findings indicate a possible functional implication of TFPIβ in locally reducing TF signaling and breast cancer cell progression, further endorsing involvement of TFPI in malignant disease.

## Materials and methods

### Reagents

RPMI1640, Dulbecco’s modified Eagle’s medium (DMEM), Endothelial Basal Medium-2 (EBM-2), EGM-2-MV SingleQuots, fetal bovine serum (FBS) and phosphate buffered saline (PBS) were purchased from Lonza (Viviere, Belgium), while the Human mammary epithelial cell (HuMEC) ready medium, Alexa Fluor® 488 goat anti-rabbit IgG (H+L) antibody (A-11008), and *SlowFade*® Gold antifade reagent w/DAPI (S36938) were from Life Technologies (Carlsbad, CA, USA). The polyclonal rabbit anti-human tissue factor pathway inhibitor antibody (4901/ADG72), monoclonal mouse anti-human tissue factor pathway inhibitor antibody (4903), and monoclonal mouse anti-human tissue factor antibody (ADG4508) were from American Diagnostica (Greenwich, CT, USA), while the rabbit-IgG-UNLB and goat anti-rabbit IgG (H+L)-RPE antibodies were from Southern Biotechnology Associates (Birmingham, AL, USA). The human γ-globulin antibody, PI-PLC, and formalin solution (4% formaldehyde, HT5011) were from Sigma-Aldrich (St. Louis, MO, USA). Heparin was purchased from Leo Pharma (Ballerud, Denmark). The RNaqueous kit (AM1912), High Capacity cDNA Reverse Transcription Kit, TaqMan Gene Expression Master Mix, TaqMan TF assay (Hs00175225_m1), and pre-developed TaqMan Human 18S rRNA assay were all from Applied Biosystems (Life Technologies). Purified bovine FX, FXa, and FXa chromogenic substrate CS-11(22) were from Aniara Diagnostica (Mason, OH, USA), while recombinant FVIIa was from Novo Nordisk AS (Bagsvaerd, Denmark).

### Cell cultures

The human mammary adenocarcinoma cell lines SK-BR-3 (ATCC HTB-30) and MCF-7 (ATCC HTB-22) and the human mammary ductal carcinoma cell lines ZR-75-1 (ATCC CRL-1500) and BT-474 (ATCC HTB-20) were grown in RPMI1640 with phenol red and 2 mM L-glutamine supplemented with 10% heat inactivated FBS. The intraductal carcinoma cell lines Sum102 and Sum149 and the transformed breast epithelial cell line ME16C2 (hTERT-HME1, ATCC CRL-4010) were grown in HuMEC ready medium containing HuMEC supplements (epidermal growth factor, hydrocortisone, isoprotenerol, transferrin and insulin) and Bovine Pituitary Extract. 5% heat-inactivated FBS was included in the growth medium of Sum149 cells. The human mammary adenocarcinoma cell line MDA-MB-231 (ATCC HTB-26), the human endothelial cell line EA.hy926 (ATCC CRL-2922), and the Chinese hamster ovary cell line CHO-K1 (ATCC CCL-61) were cultured in DMEM containing 2 mM L-glutamine, 4.5 g/L glucose and 10% heat inactivated FBS. The primary human coronary artery endothelial cells HCAEC (#CC-2585, Lonza) were cultivated in modified EBM-2 basal medium supplemented with EGM-2-MV SingleQuots (containing vascular endothelial growth factor, basic fibroblast growth factor, insulin-like growth factor-I, epidermal growth factor, ascorbic acid, and gentamicin), and 10% FBS. All cells were cultured at 37°C in an incubator with a humidified atmosphere and 5% CO_2_.

### Quantitative real-time PCR

Total RNA was isolated from the cells using the RNaqueous kit according to the manufacturer’s protocol, and the concentration of the isolated RNA was assessed by the NanoDrop1 ND-1000 UV–vis Spectrophotometer (NanoDrop Technologies, Wilmington, DE, USA). cDNA was synthesized from total RNA using the High Capacity cDNA Reverse Transcription Kit, and 19 ng of cDNA were PCR amplified on the ABI PRISM 7900 Sequence Detection System (Applied Biosystems) according to the protocol, using the TaqMan Gene Expression Master Mix, and the self-designed TFPIα and TFPIβ specific assays [21] or a Taqman TF assay. All samples were run in triplicate. The relative mRNA expression was calculated using the comparative Ct method, normalizing the Ct values against the endogenous control 18S rRNA, and using HCAEC expression as a normal endothelial reference and CHO expression values as a negative sample. The 18S rRNA showed the least expression variation between the different cell lines of the endogenous controls tested.

### TFPI and TF antigen

To determine TFPI and TF protein expression, cells were seeded in six-well trays and grown for three days until ~80% confluence before harvesting. Media were collected and cells were lysed as previously described [20]. The commercial ELISA kits Free and Total TFPI (Asserachrom®, Diagnostica Stago, Asnière, France) were used to measure TFPI antigen in the cell media/lysate, and Zymutest Tissue Factor full length (RK035A, Hyphen BioMed) to measure full length TF in cell lysates, according to the manufacturers’ protocols. The results were corrected against cellular total protein, as measured in cell lysates by the modified Lowry assay (Bio-Rad *D*_*C*_ Protein Assay, Bio-Rad Laboratories, Hercules, CA, USA).

### PI-PLC and heparin treatment

Confluent cells in six-wells trays were washed three times with PBS and serum starved for one hour before treated with PI-PLC (1 U/mL), or heparin (5 U/mL) for two hours, or with PI-PLC for two hours followed by removal of the supernatant and subsequent treatment with heparin for additional two hours, at 37°C. Serum-free media (SFM) was used as a control for the treatments. A second control containing SFM and glycerol from a 55% solution with 0.05% NaN3 to mimic the PI-PLC buffer was also tested but showed no differences from SFM alone. The supernatants were collected and analyzed for TFPI antigen, while the cells were washed twice with PBS and collected for flow cytometry analysis or lysed as previously described [20] for antigen detection.

### Flow cytometry

Immediately after treatment, Sum 102, MDA-MB-231, and HCAEC cells were detached using 5 mM EDTA and transferred to Eppendorf tubes. Cells were washed twice in cold PBS before blocked with human γ-globulin (100 μg/mL) in dilution buffer (PBS with 1% BSA and 12.5 mM Na-Azide) for 15–20 min. Cells were incubated with a rabbit anti-human TFPI specific antibody or an irrelevant rabbit antibody at a final concentration of 40 ng/μl for 30 min at 4°C. Cells were washed twice in cold PBS and stained with RPE linked goat anti-rabbit antibody for 30 min at 4°C, before pelleted cells were resuspended in dilution buffer. Flow cytometry analysis was performed on the FACSCalibur flow cytometer (Becton Dickinson, Heidelberg, Germany), and the CellQuest 3.3 software (BD Biosciences) was used for data collection. Flow data were analyzed and presented using Flow Jo 7.6.4 (Tree Star, Inc., Ashland, OR).

### Immunofluorescence microscopy

Sum102 cells (1.0⋅10^5^/well) were seeded the day before in 4-wells glass chamber slides (Lab Tek II, Thermo Fisher Scientific, Waltham, MA), serum-starved for 1 hour and treated with SFM containing glycerol from a 55% solution with 0.05% NaN3 (denoted untreated) or PI-PLC (1 U/mL) for 2 hours at 37°C. After treatment, the cells were washed once in PBS, fixed with formalin for 20 min on ice, washed once with PBS (with 1% FBS) and incubated 20 min at RT with blocking buffer (PBS with 5% BSA and 1% Tween 20). After blocking, the cells were washed once in dye buffer (PBS with 1% BSA and 1% Tween 20) and incubated with primary rabbit anti-human TFPI and mouse anti-human TF specific antibodies for 45 min at RT. Cells were washed three times with dye buffer and stained with fluorescent goat anti-rabbit and donkey anti-mouse secondary antibodies for 45 min at RT. The cells were washed three times with dye buffer, the chambers were released from the slides, and two drops of antifade solution were added and the slides sealed with cover glass. Stained cells were visualized using a fluorescence Nikon eclipse E400 inverted microscope with a Plan Fluor 40x/0.75 DIC M objective (Nikon, Tokyo, Japan) and the appropriate filter. Images were captured using a Nikon digital sight DS-Fi1 Camera system.

### TF-FVIIa activity

Sum102 (1.5⋅10^5^/well), MDA-MB-231 (0.5⋅10^5^/well), and HCAEC (0.7⋅10^5^/well) cells were seeded the day prior to the experiment, serum-starved for 1 hour and treated with SFM (denoted untreated), PI-PLC (1 U/mL), TFPI blocking antibody (10 μg/mL), or TF blocking antibody (10/20 μg/mL) for 2 hours at 37°C. After treatment, the cells were washed twice in wash solution (10 mM HEPES, 150 mM NaCl, 4 mM KCl, and 11 mM Glucose, pH 7.5) and incubated for 1 hour at 37°C in reaction solution (wash buffer with 5 mg/mL BSA and 5 mM CaCl_2_, pH 7.5) containing 10 nM FVIIa and 175 nM FX. Following incubation, 50 μL were transferred to 100 μL stop solution (50 mM Tris HCl, 150 mM NaCl, 25 mM EDTA, and 1 mg/mL BSA, pH 7.5) on ice before incubated with 50 μL CS-11(22) substrate. The absorbance at 405 nm was recorded at 37°C for 45 min at 15 sec intervals using a Spectra Max Plus 384 microplate reader (Molecular Devices, Sunnyvale, CA, USA). The maximum velocities (V_max_) in mU/min were used to calculate the amount of FXa generated, using a standard curve obtained with known concentrations of FXa.

### Western blotting

Supernatants from Sum102 cells treated with SFM containing glycerol from a 55% solution with 0.05% NaN3 (denoted untreated) or PI-PLC were collected and deglycosylated using the Enzymatic Protein Deglycosylation Kit (Sigma-Aldrich) following the manufacturer’s instruction, combined with loading buffer (Bio-Rad Laboratories Hercules, CA) containing 5% β-mercaptoethanol and denatured for 5 min at 97°C. The concentrated supernatants were separated on a 10% SDS-polyacrylamide gel (Bio-Rad), before proteins were transferred to a PVDF membrane (Bio-Rad), blocked in 5% BSA, and incubated with a primary anti-human TFPI specific antibody over night at 4°C under constant agitation. After incubation with the appropriate secondary HRP-linked antibody for 1 hour at 20°C, proteins were visualized using the ECL Plus Western Blotting Detection System (GE Healthcare, Buckinghamshire, UK). Images were produced using the Luminescent Image Analyzer LAS-4000 mini (Fujifilm, Tokyo, Japan).

### Statistical analysis

The associations between mRNA and protein levels were evaluated using Pearsons correlation test, while significant differences between treated samples and controls were calculated using the Student’s t or one-way ANOVA (Bonferroni corrected) tests in GraphPad Prism 5.0 (Graphpad, San Diego, CA, USA), and a *P*-value of <.05 was considered statistically significant. * = *p*<.05, ** = *p*<.01, and *** = *p*<.001.

## Abbreviations

CHO-K1: Chinese hamster ovary cell line; DMEM: Dulbecco’s Modified Eagle’s Medium; ELISA: Enzyme-linked immunoabsorbent assay; EBM/EGM: Endothelial Basal/Growth Medium; FBS: Fetal bovine serum; FVIIa/FXa: Factor VIIa/FXa; GPI: Glycosylphosphatidylinositol; HCAEC: Human coronary artery endothelial cells; HuMEC: Human mammary epithelial cells; PBS: Phosphate buffered saline; PI-PLC: Phosphatidylinositol-phospholipase C; SFM: Serum-free media; TF: Tissue factor; TFPI: Tissue factor pathway inhibitor.

## Competing interests

The authors declare that they have no competing interests.

## Authors’ contributions

MS, MT, and BS performed the experiments; MT and BS designed the research, analyzed the results and wrote the paper; GS, PMS and NI conceived the project, interpreted results and edited the paper; NI designed the research. All authors read and approved the final manuscript.
